# Benzyl 2-(benzylsulfanyl)benzoate

**DOI:** 10.1107/S1600536813001761

**Published:** 2013-01-23

**Authors:** Zorica Leka, Sladjana B. Novaković, Goran A. Bogdanović, Gordana P. Radić, Zoran R. Ratković

**Affiliations:** a Faculty of Metallurgy and Technology, University of Montenegro, Cetinjski put bb, 81000 Podgorica, Montenegro; b Vinča Institute of Nuclear Sciences, Laboratory of Theoretical Physics and Condensed Matter Physics, University of Belgrade, PO Box 522, 11001 Belgrade, Serbia; c Faculty of Medical Sciences, University of Kragujevac, S. Markovića 69, 34000 Kragujevac, Serbia; d Department of Chemistry, Faculty of Science, University of Kragujevac, R. Domanovića 12, 34000 Kragujevac, Serbia

## Abstract

In the title compound, C_21_H_18_O_2_S, the central aromatic ring makes dihedral angles of 5.86 (12) and 72.02 (6)° with the rings of the terminal *O*-benzyl and *S*-benzyl groups, respectively. The dihedral angle between the peripheral phenyl rings is 66.16 (6)°. In the crystal, mol­ecules are linked by pairs of C—H⋯O hydrogen bonds, forming inversion dimers. These dimers are linked *via* C—H⋯π inter­actions, forming a three-dimensional network.

## Related literature

For related structures, see: Radić *et al.* (2012 ▶); Lucena *et al.* (1996 ▶); Sillanpää *et al.* (1994 ▶); Alhadi *et al.* (2010 ▶). For the biological activity of thio­salicylic acid derivatives, see: Bernardelli *et al.* (2005 ▶); Halaschek-Wiener *et al.* (2003 ▶); Sadao *et al.* (2000 ▶).
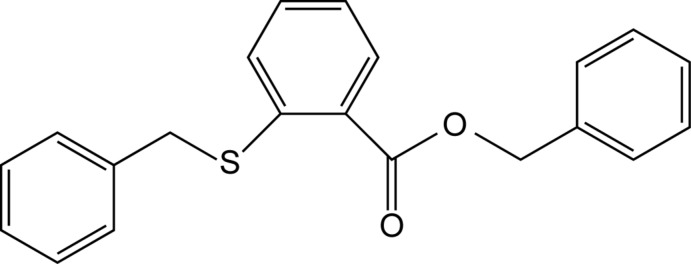


## Experimental

### 

#### Crystal data

C_21_H_18_O_2_S
*M* *_r_* = 334.41
Triclinic, 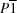

*a* = 5.6957 (3) Å
*b* = 12.1117 (11) Å
*c* = 13.0813 (11) Å
α = 72.748 (8)°
β = 86.477 (6)°
γ = 89.941 (6)°
*V* = 860.04 (12) Å^3^
*Z* = 2
Cu *K*α radiation
μ = 1.74 mm^−1^
*T* = 293 K
0.11 × 0.10 × 0.05 mm

#### Data collection

Oxford Diffraction Xcalibur Gemini Sapphire3 diffractometer
Absorption correction: multi-scan (*CrysAlis PRO*; Oxford Diffraction, 2012 ▶) *T*_min_ = 0.944, *T*_max_ = 1.000
5368 measured reflections
3299 independent reflections
2554 reflections with *I* > 2σ(*I*)
*R*_int_ = 0.018

#### Refinement

*R*[*F* ^2^ > 2σ(*F* ^2^)] = 0.040
*wR*(*F* ^2^) = 0.113
*S* = 1.04
3299 reflections
217 parameters
H-atom parameters constrained
Δρ_max_ = 0.21 e Å^−3^
Δρ_min_ = −0.21 e Å^−3^

### 

Data collection: *CrysAlis PRO* (Oxford Diffraction, 2012 ▶); cell refinement: *CrysAlis PRO*; data reduction: *CrysAlis PRO*; program(s) used to solve structure: *SHELXS97* (Sheldrick, 2008 ▶); program(s) used to refine structure: *SHELXL97* (Sheldrick, 2008 ▶); molecular graphics: *ORTEP-3 for Windows* (Farrugia, 2012 ▶) and *Mercury* (Macrae *et al.*, 2006 ▶); software used to prepare material for publication: *WinGX* (Farrugia, 2012 ▶), *PLATON* (Spek, 2009) ▶ and *PARST* (Nardelli, 1995 ▶).

## Supplementary Material

Crystal structure: contains datablock(s) I, global. DOI: 10.1107/S1600536813001761/rz5036sup1.cif

Structure factors: contains datablock(s) I. DOI: 10.1107/S1600536813001761/rz5036Isup2.hkl

Supplementary material file. DOI: 10.1107/S1600536813001761/rz5036Isup3.cml

Additional supplementary materials:  crystallographic information; 3D view; checkCIF report

## Figures and Tables

**Table 1:** Geometry of hydrogen bonds and weak C—H⋯π interactions (Å, °) *Cg*1, *Cg*2 and *Cg*3 are the centroids of the C2–C7, C9–C14 and C16–C21 rings, respectively.

*D*—H⋯*A*	*D*—H	H⋯*A*	*D*⋯*A*	*D*—H⋯*A*
C14—H14⋯O2^i^	0.93	2.63	3.408 (3)	142
C4—H4⋯*Cg*3^ii^	0.93	3.11	3.846 (3)	137
C13—H13⋯*Cg*3^i^	0.93	2.88	3.653 (3)	141
C18—H18⋯*Cg*1^iii^	0.93	3.20	3.820 (3)	126
C20—H20⋯*Cg*2^iv^	0.93	2.96	3.651 (3)	132

Symmetry codes: (i) 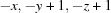; (ii) 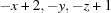; (iii) 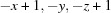; (iv) 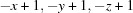.

**Fig. 1.**
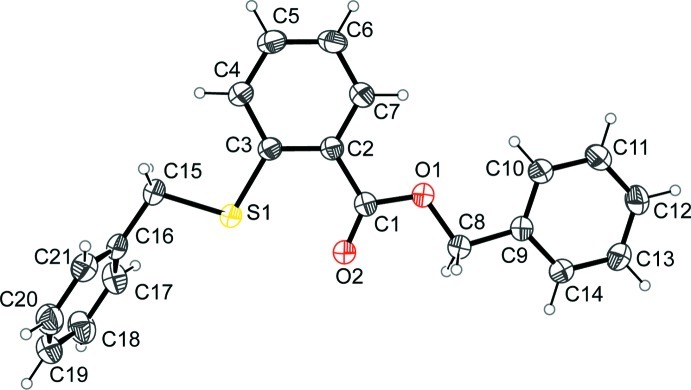
The molecular structure of the title compound with 40% probability displacement ellipsoids for non-H atoms.

**Fig. 2.**
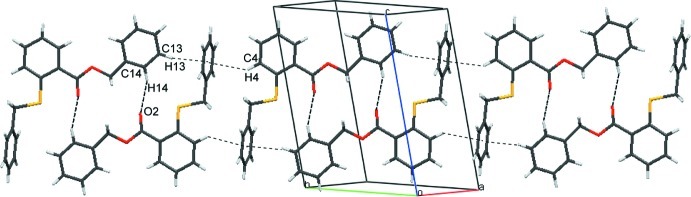
Partial crystal packing of the title compound. The C14—H···O2 and C13—H···π connected pairs of molecules are linked into a chain by C4—H4···π interactions.

## supplementary crystallographic information

### Comment

Thiosalicylic acid and its derivatives find potential application in numerous disease treatments, in particular inflammatory, allergic and respiratory diseases (Bernardelli *et al.*, 2005) as well as Ras tumor growth inhibitors (Halaschek-Wiener *et al.*, 2003). Ketones derived from thiosalicylic acids have application as bile acid transport inhibitors (Sadao *et al.*, 2000). In continuation of our work on structural and biological properties of thiosalicylic derivatives and their metal complexes (Radić *et al.*, 2012) here we present the crystal structure of the novel ligand benzyl 2-(benzylsulfanyl)benzoate.

The bond lengths and angles in the title compound are within the expected ranges. The dihedral angle between aromatic rings of the central thiosalicylic and terminal *O*-benzyl group is 5.86 (12)° indicating a nearly co-planar orientation of these two fragments. On the other hand, the ring of the *S*-benzyl group is significantly twisted with respect to the central ring forming a dihedral angle of 72.02 (6)°. In the previously reported crystal structures with *S*-benzyl derivatives of thiosalicylic acid the analogue dihedral angle varies from 13.4 to 88.9°, suggesting the free rotation of the *S*-benzyl ring around the single bonds of the C3–S1–C15–C16 fragment. The specific property of the present compound is that the atoms S1, C15 are C16 all lie in the plane of the central ring within 0.03 Å. In the cases of similar uncoordinated (Sillanpää *et al.*, 1994; Alhadi *et al.*, 2010] as well as coordinated ligands (Radić *et al.*, 2012; Lucena *et al.*, 1996) the corresponding atom C16 is significantly out of the central ring plane (1.57 Å in average).

The pair of C14—H14···O2 interactions connects inversion related molecules into dimers (Table 1, Fig. 2). This connection is reinforced by means of C—H···π interactions involving the *O*-benzyl C13—H13 donor and the *S*-benzyl ring from an inversion-related molecule as acceptor (Table 1; *Cg*3 is the centroid of the *S*-benzyl ring). The *S*-benzyl ring also serves as a π-acceptor for C—H donors of a neighboring molecular pair; in that way the pairs of molecules further arrange into chain by C4— H4···*Cg*3 interactions (Table 1, Fig. 2). The molecules are further associated into a three-dimensional structure by C—H···π interactions which engage two remaining phenyl rings as π-acceptors (Table 1, *Cg*1 and *Cg*2 are centroids of central thiosalicylic and terminal *O*-benzyl rings, respectively).

### Experimental

The thioacid ligand was prepared by alkylation of thiosalicylic acid by means of the corresponding alkyl halogenides in alkaline water-ethanol solution. Thiosalicylic acid (1 mmol) was added to a 100 cm^3^ round bottom flask containing 50 cm^3^ of a 30% solution of ethanol in water and stirred. A solution of NaOH (2 mmol in 5 cm^3^ of water) was added to the acid suspension, whereupon the solution became clear. The corresponding benzyl halogenide (2 mmol) was dissolved in 5 cm^3^ of ethanol and transferred to the stirred solution. The resulting mixture was kept overnight at 60°C. The reaction mixture was transffered into a beaker and ethanol was evaporated off on a water bath. Diluted hydrochloric acid (2 mol/dm^3^) was added to the resulting water solution and *S*-benzyl thiosalicylic acid was precipitated as a white powder. The obtained acid was filtered off and washed with plenty of distilled water. The product was dried under vacuum overnight. Crystals of the title compound suitable for X-ray determination were obtained by slow evaporation of a benzyl alcohol solution, which unexpectedly resulted in the esterification of the carboxylic group of thiosalicylic acid.

### Refinement

H atoms bonded to C atoms were placed at geometrically calculated positions and refined using a riding model. C—H distances were fixed to 0.93 and 0.97 Å from aromatic and methylene C atoms, respectively. The *U*_iso_(H) values were equal to 1.2 times *U*_eq_ of the corresponding aromatic C(*sp*^2^) and methylene C(*sp*^3^) atoms.

### Crystal data

**Table:**

C_21_H_18_O_2_S	*Z* = 2
*M**_r_* = 334.41	*F*(000) = 352
Triclinic, *P*1	*D*_x_ = 1.291 Mg m^−^^3^
Hall symbol: -P 1	Cu *K*α radiation, λ = 1.54180 Å
*a* = 5.6957 (3) Å	Cell parameters from 2123 reflections
*b* = 12.1117 (11) Å	θ = 3.5–72.3°
*c* = 13.0813 (11) Å	µ = 1.74 mm^−^^1^
α = 72.748 (8)°	*T* = 293 K
β = 86.477 (6)°	Prism, colourless
γ = 89.941 (6)°	0.11 × 0.10 × 0.05 mm
*V* = 860.04 (12) Å^3^	

### Data collection

**Table:**

Oxford Diffraction Xcalibur Gemini Sapphire3 diffractometer	3299 independent reflections
Radiation source: Enhance (Cu) X-ray Source	2554 reflections with *I* > 2σ(*I*)
Graphite monochromator	*R*_int_ = 0.018
Detector resolution: 16.3280 pixels mm^-1^	θ_max_ = 72.4°, θ_min_ = 3.6°
ω scans	*h* = −5→7
Absorption correction: multi-scan (*CrysAlis PRO*; Oxford Diffraction, 2012)	*k* = −14→14
*T*_min_ = 0.944, *T*_max_ = 1.000	*l* = −16→15
5368 measured reflections	

### Refinement

**Table:**

Refinement on *F*^2^	Primary atom site location: structure-invariant direct methods
Least-squares matrix: full	Secondary atom site location: difference Fourier map
*R*[*F*^2^ > 2σ(*F*^2^)] = 0.040	Hydrogen site location: inferred from neighbouring sites
*wR*(*F*^2^) = 0.113	H-atom parameters constrained
*S* = 1.04	*w* = 1/[σ^2^(*F*_o_^2^) + (0.0493*P*)^2^ + 0.0651*P*] where *P* = (*F*_o_^2^ + 2*F*_c_^2^)/3
3299 reflections	(Δ/σ)_max_ < 0.001
217 parameters	Δρ_max_ = 0.21 e Å^−^^3^
0 restraints	Δρ_min_ = −0.21 e Å^−^^3^

### Special details

**Table:**

Experimental. IR (KBr, cm^-1^): 3414, 3061, 2920, 2648, 2559, 1674, 1584, 1562, 1463, 1412, 1317, 1272, 1255, 1154, 1062, 1046, 897, 743, 711, 652, 551. ^1^H NMR (200 MHz, CDCl_3_, δ p.p.m.): 4.11 (s, 2H, C^15^H2), 5.45 (s, 2H, C^8^H_2_), 7.00–7.93 (m, 17H, Ar and bz). ^13^C NMR (50 MHz, DMSO-d6, δ p.p.m.): 38.5 (C^15^H2), 68.3 (C^8^H2) 125.0; 126.3; 126.8; 127.0; 127.2; 127.5; 127.8; 127.9; 128.1; 128.3; 128.8; 128.9, 129.3; 130.2; 133.5; 139.5; 141.7; 142.3 (Ar and bz), 166.8 (COObz).

### Fractional atomic coordinates and isotropic or equivalent isotropic displacement parameters (Å^2^)

**Table:**

	*x*	*y*	*z*	*U*_iso_*/*U*_eq_	
S1	0.60616 (9)	0.22258 (5)	0.48163 (4)	0.06178 (18)	
O1	0.1278 (2)	0.47589 (12)	0.28903 (10)	0.0595 (4)	
O2	0.2518 (3)	0.37675 (15)	0.44697 (11)	0.0859 (6)	
C1	0.2696 (3)	0.40163 (17)	0.35128 (15)	0.0549 (5)	
C2	0.4509 (3)	0.35644 (16)	0.28855 (14)	0.0519 (4)	
C3	0.6149 (3)	0.27491 (16)	0.34054 (14)	0.0519 (4)	
C4	0.7850 (4)	0.23806 (19)	0.27595 (16)	0.0632 (5)	
H4	0.8945	0.1838	0.3081	0.076*	
C5	0.7919 (4)	0.2808 (2)	0.16621 (17)	0.0728 (6)	
H5	0.9072	0.2555	0.1253	0.087*	
C6	0.6324 (4)	0.3601 (2)	0.11575 (17)	0.0743 (6)	
H6	0.6388	0.3885	0.0413	0.089*	
C7	0.4622 (4)	0.39694 (19)	0.17718 (15)	0.0650 (5)	
H7	0.3525	0.4501	0.1433	0.078*	
C8	−0.0440 (4)	0.53023 (19)	0.34328 (15)	0.0602 (5)	
H8A	0.0348	0.5751	0.3817	0.072*	
H8B	−0.1426	0.4718	0.3946	0.072*	
C9	−0.1923 (3)	0.60777 (16)	0.26112 (15)	0.0522 (4)	
C10	−0.1380 (4)	0.6363 (2)	0.15204 (16)	0.0659 (6)	
H10	−0.0031	0.6073	0.1264	0.079*	
C11	−0.2821 (4)	0.7076 (2)	0.08082 (17)	0.0725 (6)	
H11	−0.2437	0.7260	0.0075	0.087*	
C12	−0.4810 (4)	0.7514 (2)	0.11702 (18)	0.0712 (6)	
H12	−0.5780	0.7990	0.0686	0.085*	
C13	−0.5362 (4)	0.7247 (2)	0.22537 (18)	0.0678 (6)	
H13	−0.6698	0.7552	0.2505	0.081*	
C14	−0.3935 (3)	0.65252 (18)	0.29709 (16)	0.0592 (5)	
H14	−0.4332	0.6339	0.3703	0.071*	
C15	0.8417 (4)	0.11867 (19)	0.50644 (16)	0.0637 (5)	
H15A	0.8106	0.0571	0.4754	0.076*	
H15B	0.9904	0.1561	0.4746	0.076*	
C16	0.8515 (3)	0.07081 (16)	0.62584 (15)	0.0555 (5)	
C17	0.6840 (4)	−0.0071 (2)	0.68695 (18)	0.0715 (6)	
H17	0.5632	−0.0315	0.6536	0.086*	
C18	0.6927 (4)	−0.0493 (2)	0.79649 (19)	0.0817 (7)	
H18	0.5787	−0.1022	0.8364	0.098*	
C19	0.8689 (4)	−0.0138 (2)	0.84739 (19)	0.0757 (6)	
H19	0.8743	−0.0418	0.9215	0.091*	
C20	1.0364 (4)	0.0634 (2)	0.78757 (19)	0.0746 (6)	
H20	1.1571	0.0872	0.8214	0.090*	
C21	1.0283 (4)	0.10629 (19)	0.67754 (17)	0.0641 (5)	
H21	1.1423	0.1594	0.6380	0.077*	

### Atomic displacement parameters (Å^2^)

**Table:**

	*U*^11^	*U*^22^	*U*^33^	*U*^12^	*U*^13^	*U*^23^
S1	0.0688 (3)	0.0641 (3)	0.0475 (3)	0.0269 (2)	−0.0004 (2)	−0.0098 (2)
O1	0.0647 (8)	0.0623 (8)	0.0502 (7)	0.0208 (7)	−0.0064 (6)	−0.0145 (6)
O2	0.0995 (12)	0.1023 (13)	0.0475 (8)	0.0567 (10)	−0.0012 (7)	−0.0103 (8)
C1	0.0607 (11)	0.0530 (11)	0.0481 (10)	0.0107 (9)	−0.0047 (8)	−0.0106 (8)
C2	0.0601 (11)	0.0465 (10)	0.0469 (10)	0.0058 (8)	0.0000 (8)	−0.0111 (8)
C3	0.0570 (11)	0.0489 (10)	0.0483 (10)	0.0060 (8)	0.0028 (8)	−0.0136 (8)
C4	0.0660 (12)	0.0638 (13)	0.0567 (11)	0.0154 (10)	0.0050 (9)	−0.0152 (9)
C5	0.0790 (15)	0.0769 (15)	0.0615 (13)	0.0124 (12)	0.0148 (10)	−0.0229 (11)
C6	0.0987 (17)	0.0748 (15)	0.0450 (11)	0.0102 (13)	0.0089 (11)	−0.0139 (10)
C7	0.0794 (14)	0.0618 (13)	0.0501 (11)	0.0136 (11)	−0.0039 (10)	−0.0109 (9)
C8	0.0645 (12)	0.0638 (12)	0.0502 (11)	0.0173 (10)	−0.0028 (9)	−0.0138 (9)
C9	0.0542 (10)	0.0489 (10)	0.0523 (10)	0.0059 (8)	−0.0050 (8)	−0.0129 (8)
C10	0.0618 (12)	0.0783 (15)	0.0537 (11)	0.0165 (11)	−0.0007 (9)	−0.0142 (10)
C11	0.0755 (14)	0.0837 (16)	0.0506 (11)	0.0134 (12)	−0.0052 (10)	−0.0077 (10)
C12	0.0655 (13)	0.0764 (15)	0.0673 (14)	0.0172 (11)	−0.0186 (10)	−0.0120 (11)
C13	0.0591 (12)	0.0716 (14)	0.0738 (14)	0.0181 (10)	−0.0061 (10)	−0.0228 (11)
C14	0.0609 (12)	0.0636 (12)	0.0546 (11)	0.0099 (10)	−0.0031 (9)	−0.0202 (9)
C15	0.0637 (12)	0.0635 (12)	0.0621 (12)	0.0257 (10)	−0.0048 (9)	−0.0160 (10)
C16	0.0553 (11)	0.0490 (10)	0.0615 (12)	0.0180 (9)	−0.0052 (9)	−0.0150 (9)
C17	0.0682 (13)	0.0626 (13)	0.0766 (15)	0.0004 (11)	−0.0176 (11)	−0.0073 (11)
C18	0.0813 (16)	0.0699 (15)	0.0762 (16)	−0.0043 (12)	−0.0062 (12)	0.0059 (12)
C19	0.0878 (17)	0.0730 (15)	0.0603 (13)	0.0167 (13)	−0.0134 (12)	−0.0091 (11)
C20	0.0734 (15)	0.0783 (16)	0.0769 (15)	0.0095 (12)	−0.0201 (12)	−0.0272 (13)
C21	0.0563 (12)	0.0637 (13)	0.0717 (14)	0.0039 (10)	−0.0022 (10)	−0.0197 (10)

### Geometric parameters (Å, º)

**Table:**

S1—C3	1.7623 (19)	C10—H10	0.9300
S1—C15	1.8159 (18)	C11—C12	1.368 (3)
O1—C1	1.331 (2)	C11—H11	0.9300
O1—C8	1.444 (2)	C12—C13	1.373 (3)
O2—C1	1.195 (2)	C12—H12	0.9300
C1—C2	1.483 (3)	C13—C14	1.382 (3)
C2—C7	1.390 (2)	C13—H13	0.9300
C2—C3	1.410 (3)	C14—H14	0.9300
C3—C4	1.405 (2)	C15—C16	1.501 (3)
C4—C5	1.372 (3)	C15—H15A	0.9700
C4—H4	0.9300	C15—H15B	0.9700
C5—C6	1.371 (3)	C16—C17	1.379 (3)
C5—H5	0.9300	C16—C21	1.383 (3)
C6—C7	1.379 (3)	C17—C18	1.375 (3)
C6—H6	0.9300	C17—H17	0.9300
C7—H7	0.9300	C18—C19	1.375 (3)
C8—C9	1.504 (2)	C18—H18	0.9300
C8—H8A	0.9700	C19—C20	1.368 (3)
C8—H8B	0.9700	C19—H19	0.9300
C9—C10	1.380 (3)	C20—C21	1.381 (3)
C9—C14	1.383 (3)	C20—H20	0.9300
C10—C11	1.379 (3)	C21—H21	0.9300
			
C3—S1—C15	103.19 (9)	C12—C11—H11	119.7
C1—O1—C8	116.17 (14)	C10—C11—H11	119.7
O2—C1—O1	122.68 (18)	C11—C12—C13	119.5 (2)
O2—C1—C2	124.83 (17)	C11—C12—H12	120.2
O1—C1—C2	112.48 (16)	C13—C12—H12	120.2
C7—C2—C3	119.66 (17)	C12—C13—C14	120.1 (2)
C7—C2—C1	119.51 (17)	C12—C13—H13	120.0
C3—C2—C1	120.82 (16)	C14—C13—H13	120.0
C4—C3—C2	117.65 (17)	C13—C14—C9	120.71 (19)
C4—C3—S1	121.64 (15)	C13—C14—H14	119.6
C2—C3—S1	120.71 (14)	C9—C14—H14	119.6
C5—C4—C3	121.03 (19)	C16—C15—S1	107.11 (13)
C5—C4—H4	119.5	C16—C15—H15A	110.3
C3—C4—H4	119.5	S1—C15—H15A	110.3
C6—C5—C4	121.3 (2)	C16—C15—H15B	110.3
C6—C5—H5	119.4	S1—C15—H15B	110.3
C4—C5—H5	119.4	H15A—C15—H15B	108.5
C5—C6—C7	118.9 (2)	C17—C16—C21	118.3 (2)
C5—C6—H6	120.5	C17—C16—C15	121.5 (2)
C7—C6—H6	120.5	C21—C16—C15	120.2 (2)
C6—C7—C2	121.5 (2)	C18—C17—C16	121.0 (2)
C6—C7—H7	119.3	C18—C17—H17	119.5
C2—C7—H7	119.3	C16—C17—H17	119.5
O1—C8—C9	108.69 (15)	C19—C18—C17	120.4 (2)
O1—C8—H8A	110.0	C19—C18—H18	119.8
C9—C8—H8A	110.0	C17—C18—H18	119.8
O1—C8—H8B	110.0	C20—C19—C18	119.1 (2)
C9—C8—H8B	110.0	C20—C19—H19	120.4
H8A—C8—H8B	108.3	C18—C19—H19	120.4
C10—C9—C14	118.49 (17)	C19—C20—C21	120.8 (2)
C10—C9—C8	123.42 (17)	C19—C20—H20	119.6
C14—C9—C8	118.09 (17)	C21—C20—H20	119.6
C11—C10—C9	120.55 (19)	C20—C21—C16	120.4 (2)
C11—C10—H10	119.7	C20—C21—H21	119.8
C9—C10—H10	119.7	C16—C21—H21	119.8
C12—C11—C10	120.6 (2)		

### Hydrogen-bond geometry (Å, º)

Cg1, Cg2 and Cg3 are the centroids of the C2–C7, C9–C14 and C16–C21 rings, respectively.

**Table:**

*D*—H···*A*	*D*—H	H···*A*	*D*···*A*	*D*—H···*A*
C14—H14···O2^i^	0.93	2.63	3.408 (3)	142
C4—H4···*Cg*3^ii^	0.93	3.11	3.846 (3)	137
C13—H13···*Cg*3^i^	0.93	2.88	3.653 (3)	141
C18—H18···*Cg*1^iii^	0.93	3.20	3.820 (3)	126
C20—H20···*Cg*2^iv^	0.93	2.96	3.651 (3)	132

Symmetry codes: (i) −*x*, −*y*+1, −*z*+1; (ii) −*x*+2, −*y*, −*z*+1; (iii) −*x*+1, −*y*, −*z*+1; (iv) −*x*+1, −*y*+1, −*z*+1.
